# Induction of psoriatic scalp and nail lesions in a patient treated with Ofatumumab: A potential autoimmune dysregulation induced by anti‐CD 20 therapy

**DOI:** 10.1111/srt.13662

**Published:** 2024-03-21

**Authors:** Astrid Herzum, Silvia Maria Orsi, Giulia Ciccarese, Corrado Occella, Gianmaria Viglizzo

**Affiliations:** ^1^ Dermatology Department IRCCS Istituto Giannina Gaslini Genova Italy; ^2^ Department of Neuroscience Rehabilitation Ophthalmology Genetics Maternal and Child Health University of Genoa Genoa Italy; ^3^ Dermatology Unit Department of Medical and Surgical Sciences University of Foggia and Ospedali Riuniti Foggia Italy

Dear Editor,

Ofatumumab is a humanized monoclonal antibody targeting the CD20 antigen on B‐lymphocytes, that has been recently approved for the treatment of B cell malignancies and relapsing‐remitting multiple sclerosis (RR‐MS).^1^ While most patients (90%) experience no skin issues during biologic therapy, drug‐induced psoriasis is an increasingly recognized phenomenon, and has been already linked to other anti‐CD20 monoclonal antibodies (mAbs), such as Rituximab.^2^

To date, there have been no literature reports of Ofatumumab causing psoriasis.

We report the case of a 42‐year‐old woman with intense scalp desquamation and sub‐ungueal hyperkeratosis, beginning 3 months after the introduction of Ofatumumab injection therapy for RR‐MS. The patient assumed no other medications and reported no familiar nor personal history of psoriasis.

At clinical examination, a thick, hyperkeratotic asymptomatic, plaque of the scalp was evidenced, forming tufts of hair indicative of pseudotinea amiantacea. Also, distal onycholysis of the fingernails and intense subungueal hyperkeratosis were evidenced, with splinter hemorrhages at onychoscopy (Figures 1 and 2). No lesions were found on the mucosal membranes.

**FIGURE 1:**
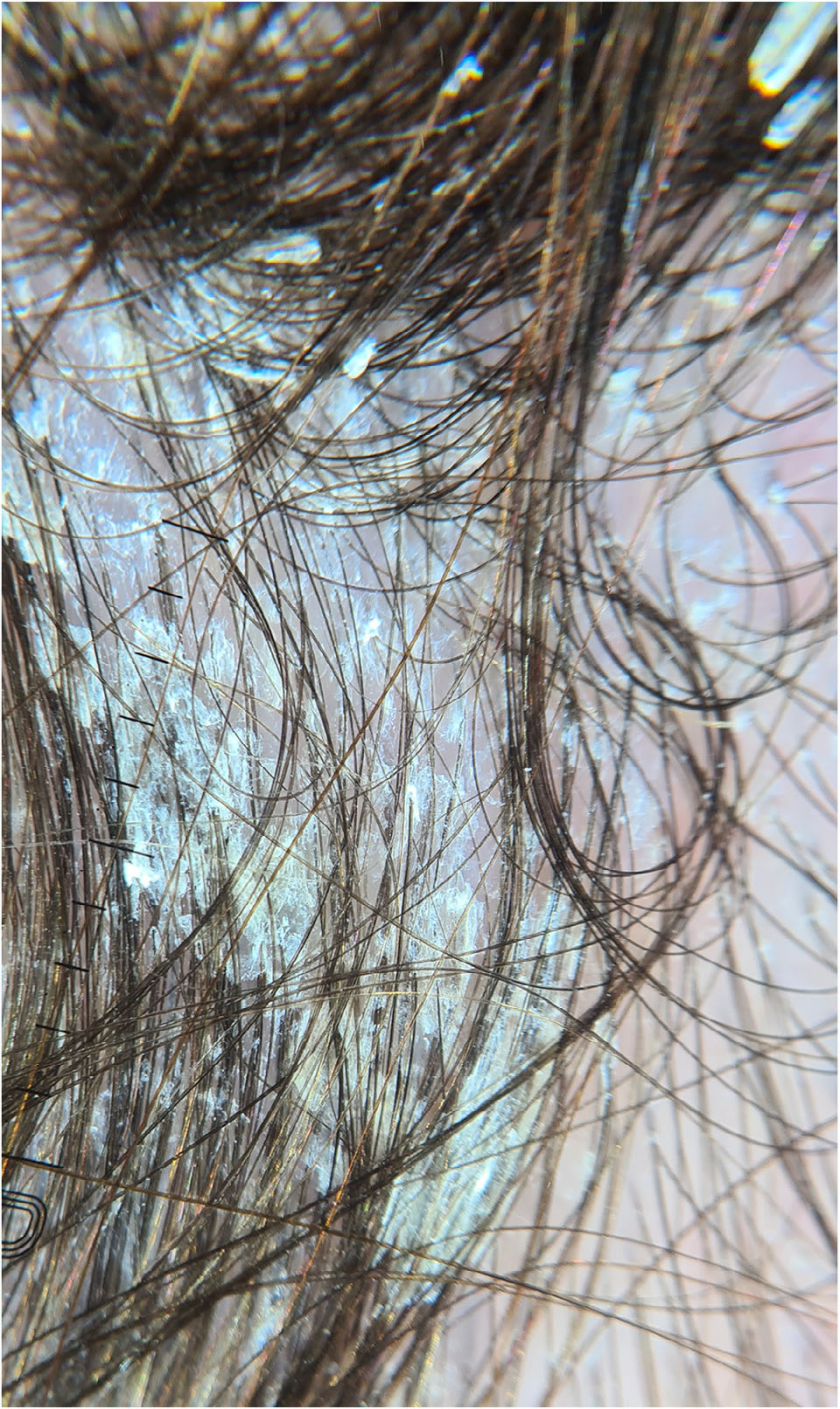
Dermoscopic image of the hyperkeratotic plaque of the scalp, with characteristic tufted hair formed by silvery scale encircling multiple hair shafts.

**FIGURE 2:**
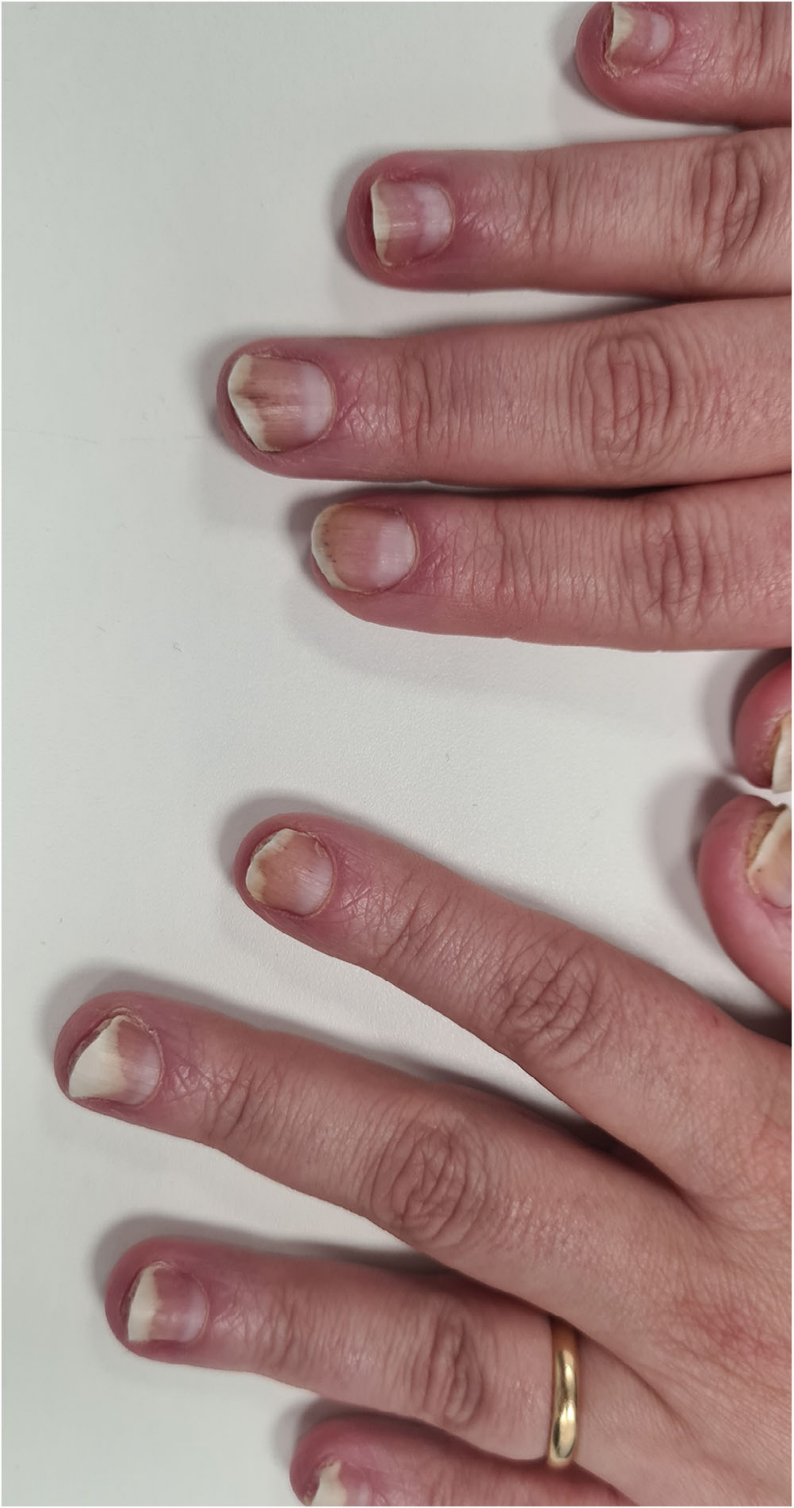
Intense subungueal hyperkeratosis of all fingernails and distal onycholysis, with splinter hemorrhages.

Routine laboratory investigations were within normal ranges, without eosinophilia, that usually suggests severe cutaneous drug eruptions.^3^

Skin and nail scraping KOH‐tests, performed to exclude fungal infections, gave negative result. The signs were consistent with a clinical diagnosis of drug‐induced psoriasis.^4^

The patient was treated with topical steroids and emollients and is currently on follow up.

Ofatumumab has already established itself as a valuable tool in the fight against B‐cell leukemia and was recently introduced for Relapsing Forms of MS under the brand name Kesimpta.^1^, ^5^ It binds specifically to the CD20 antigen, a transmembrane protein of mature B‐lymphocytes and induces their apoptosis. ^1^ Analogously to other anti‐CD 20 mAbs used for the treatment of MS, such as Rituximab, it is conceivable also for Ofatumumab to have cutaneous side effects. While most patients (90%) experience no skin issues during Rituximab therapy, some may develop reactions anywhere between 1 and 13 weeks after starting the medication. These reactions can vary in severity, with mild forms like sweating, hives, and itching being more common than severe conditions like vasculitis, SJS, TEN, paraneoplastic pemphigus, and lichenoid dermatitis.^2^ Interestingly, almost 20 cases of Rituximab‐induced psoriasis have been reported in literature, especially plaque psoriasis, with onset ranging from 2 weeks to 2 years. B‐cell depletion has been proposed to disrupt the regulatory function of B‐cells on T‐cells, potentially leading to altered immune responses, leading to T‐cell activation and changes in Th17 activity. ^6^ Another theory is that Rituximab might induce immune‐related skin lesions, caused by autoimmune phenomena.^7^ Given its similar targeting mechanism, Ofatumumab could potentially have an analogous effects to Rituximab.^8^, ^9^, ^10^

While no established link exists between Ofatumumab and psoriasis, the present report might represent an alarm bell to undergo close monitoring of treated patients, in order to identify precociously and adequately treat minor complications that might otherwise compromise the patients’ adherence to therapy. Also, further research on adverse effects of Ofatumumab might be encouraged.

## CONFLICT OF INTEREST STATEMENT

The authors declare no conflicts of interest.

## ORIGINALITY

The authors confirm the manuscript contains original, unpublished work that is not being considered for publication elsewhere

## Data Availability

The data that support the findings of this study are available on request from the corresponding author [A.H.]
